# Nasopharyngeal Pneumococcal Density during Asymptomatic Respiratory Virus Infection and Risk for Subsequent Acute Respiratory Illness

**DOI:** 10.3201/eid2511.190157

**Published:** 2019-11

**Authors:** Leigh M. Howard, Yuwei Zhu, Marie R. Griffin, Kathryn M. Edwards, John V. Williams, Ana I. Gil, Jorge E. Vidal, Keith P. Klugman, Claudio F. Lanata, Carlos G. Grijalva

**Affiliations:** Vanderbilt University Medical Center, Nashville, Tennessee, USA (L.M. Howard, Y. Zhu, M.R. Griffin, K.M. Edwards, C.F. Lanata, C.G. Grijalva);; University of Pittsburgh, Pittsburgh, Pennsylvania, USA (J.V. Williams);; Instituto de Investigacion Nutricional, Lima, Peru (A.I. Gil, C.F. Lanata);; Emory University, Atlanta, Georgia, USA (J.E. Vidal, K.P. Klugman)

**Keywords:** pneumococcus, pneumococcal colonization density, viral infection, acute respiratory illness, vaccine-preventable diseases, viruses, Peru, United States, children, respiratory infections, Streptococcus pneumoniae

## Abstract

Increased nasopharyngeal pneumococcal (*Streptococcus pneumoniae*) colonization density has been associated with invasive pneumococcal disease, but factors that increase pneumococcal density are poorly understood. We evaluated pneumococcal densities in nasopharyngeal samples from asymptomatic young children from Peru and their association with subsequent acute respiratory illness (ARI). Total pneumococcal densities (encompassing all present serotypes) during asymptomatic periods were significantly higher when a respiratory virus was detected versus when no virus was detected (p<0.001). In adjusted analyses, increased pneumococcal density was significantly associated with the risk for a subsequent ARI (p<0.001), whereas asymptomatic viral detection alone was associated with lower risk for subsequent ARI. These findings suggest that interactions between viruses and pneumococci in the nasopharynx during asymptomatic periods might have a role in onset of subsequent ARI. The mechanisms for these interactions, along with other potentially associated host and environmental factors, and their role in ARI pathogenesis and pneumococcal transmission require further elucidation.

*Streptococcus pneumoniae* (pneumococcus) is one of the most important bacterial causes of pneumonia among children and adults worldwide (*1**–**3*). Nasopharyngeal pneumococcal colonization is common in young children and represents a critical initial step in the progression to invasive disease (*4*,*5*). Increases in the density of pneumococci in the nasopharynx have been associated with the onset of respiratory illness (*6**,**7*) and might also play a role in transmission of bacteria to others (*8*). Several studies have found higher nasopharyngeal pneumococcal densities in patients with pneumonia than in healthy controls. However, no specific level of pneumococcal density has been identified that can establish the diagnosis of pneumococcal pneumonia (*9**–**11*).

The specific factors that drive increases in nasopharyngeal pneumococcal density that might lead to pneumonia or other invasive disease are not well characterized. Although an increase in density might represent expansion of a single preexisting serotype, it could also represent acquisition of a new colonizing pneumococcal serotype. New acquisition has been temporally associated with the acute onset of invasive disease (*12*,*13*). Co-detection of respiratory viruses has also been associated with increases in pneumococcal density during periods of acute respiratory illness (ARI), pneumonia, or both (*6*,*7*,*14*,*15*), suggesting that respiratory viruses and pneumococci might work synergistically in pneumonia and invasive disease pathogenesis (*16**–**20*). In addition, younger children have been shown to have higher pneumococcal densities than older children, whereas children who had received pneumococcal conjugate vaccines or lived in vaccinated communities were reported to have lower pneumococcal densities (*21*,*22*).

Respiratory viruses are frequently detected in the nasopharynx of young children during asymptomatic periods (*23**–**26*). A recent cross-sectional study of children 4–7 years of age indicated similar pneumococcal densities in asymptomatic children who had respiratory viruses detected and in children with viral upper respiratory illness (*27*). However, whether viral detection or duration of viral carriage during asymptomatic periods is associated with increases in nasopharyngeal pneumococcal density or subsequent ARI risk is unclear. This assessment requires longitudinal follow-up of individual young children. Our goal was to assess the association between viral detection and pneumococcal density during asymptomatic periods and determine whether viral–pneumococcal interactions during these periods predisposed to subsequent symptomatic ARI among young children.

## Methods

### Study Design and Setting

The Respiratory Infections in Andean Peruvian Children (RESPIRA-Peru) study is a prospective cohort study conducted in the Province of San Marcos, Department of Cajamarca, located in the northern highlands of Peru. Malaria is not endemic in this high-altitude study community (median altitude of study households 2,726 m). The population is primarily rural with low income and limited access to healthcare services, as previously described (*6*,*7*,*16*,*23*,*28*–*34*). Nearly all residents of San Marcos descended from the same ethnic group of Spanish people who mixed with the local Quechua population. During May 2009–September 2011, enrolled children <3 years of age residing in the study area were prospectively assessed for ARI symptoms during weekly household visits. An ARI episode was defined as the presence of either cough or fever. This definition of ARI has been applied in many large surveillance studies (*35*,*36*). Although fever alone is not highly specific for the diagnosis of ARI, it is well established that fever is often the only sign of ARI, especially in young children (*35*). In addition to cough and fever, we collected data on other respiratory signs and symptoms, including rhinorrhea, ear pain, malaise, tachypnea, nasal flaring, stridor, wheezing, and accessory muscle retractions. We considered a child to be asymptomatic if the child had rhinorrhea alone or no ARI symptoms. 

The 7-valent pneumococcal conjugate vaccine (PCV7) was introduced in the study region in late 2009 in a 2+1 schedule (2 primary doses at 3 and 5 months of age and a booster dose at 12 months). Very few children enrolled in 2009 had received any PCV7 doses at the time of enrollment. By the end of the study (September 2011), 62% of the study cohort had received >1 PCV7 dose (*30*). This study was approved by the Institutional Review Boards of Vanderbilt University (Nashville, Tennessee, USA) and the Instituto de Investigacion Nutricional (Lima, Peru).

### Respiratory Sample Collection and Testing

We collected nasopharyngeal swabs from each child monthly, whether or not ARI symptoms were present, and tested the swabs at Emory University (Atlanta, GA, USA) by bacterial culture for pneumococcal identification and quantitative PCR (qPCR) for pneumococcal density determinations (*6*,*7*,*16*,*29*,*31*). We also randomly selected a subset of nasopharyngeal samples collected during asymptomatic periods, >8 days apart from an ARI episode, to undergo real-time reverse transcription PCR (rRT-PCR) viral testing at Vanderbilt University for influenza virus (types A, B, and C), respiratory syncytial virus (RSV), human metapneumovirus (MPV), rhinovirus (HRV), adenovirus (AdV), and parainfluenza virus (PIV) types 1–3 (*23*,*32*). Because detections of respiratory viruses other than HRV and AdV were infrequent in nasopharyngeal samples collected during those asymptomatic periods (*23*), we classified viral detections into distinct groups: HRV only; AdV only; sole detection of other virus (influenza, RSV, MPV, or PIV); co-detection of >1 respiratory virus; and no virus detected.

### Statistical Analysis

We transformed pneumococcal density values to stabilize their variance, as previously described (*6*,*7*), by applying log_10_(*x* + 1) transformation to colonization density values, where *x* represents the measured density. We performed unadjusted group comparisons by using the Wilcoxon rank-sum test, Kruskal-Wallis test, or median test with Bonferroni adjustments, as appropriate. We used multivariable linear quantile mixed effects regression to model the median of log-transformed pneumococcal density against viral detection group, with random effects to account for correlation among multiple measurements collected from the same child. Other covariates included age at the time of sample collection, sex, recent exposure to antibiotics (i.e., aminopenicillins, cephalosporins, co-trimoxazole, chloramphenicol, or furazolidone within the previous 7 days), pneumococcal vaccination status (receipt of >1 dose of PCV7), and calendar month. Because viral infections could lead to rhinorrhea, we did not directly include this variable in our main regression model, and rhinorrhea was considered as a potential variable in the causal pathway between viral infections and pneumococcal density changes. To examine the association of pneumococcal densities during asymptomatic periods with the risk for subsequent ARI, we conducted a survival analysis by using a frailty model and accounted for repeated measurements from individual children. Because severe ARI occurred uncommonly in this household surveillance study, we did not stratify the outcome variable (ARI) by severity (*28*). For this assessment, follow-up extended from the date of an asymptomatic nasopharyngeal sample collection (>8 days after the last day of ARI symptoms) through the earliest of the following: ARI, the collection of another nasopharyngeal sample, or 60 days after collection of the initial asymptomatic nasopharyngeal sample. These censoring criteria were implemented on the basis of our monthly nasopharyngeal collection strategy and the anticipated transient duration of colonization with an individual serotype in young children, typically 6–8 weeks or less (*37**–**40*). Because viral infection or its manifestation as rhinorrhea could modify the risk for subsequent ARI (*41*), alternate models accounted for either viral infections or rhinorrhea in addition to the aforementioned covariates. We applied restricted cubic splines (RCS) to log-transformed pneumococcal densities in our frailty model to relax the assumption of linearity in our models (*42*,*43*). The estimated effects of RCS-transformed densities are presented in figures. The proportional hazards assumption of the frailty model was examined and satisfied by using a Schoenfeld’s global test. Statistical analyses were done in Stata version 14.2 (https://www.stata.com) and R version 3.5.0 (https://www.r-project.org), including lqmm and survival packages.

## Results

### Pneumococcal Colonization and Viral Detections

In total, 849 nasopharyngeal samples collected during asymptomatic periods from 480 children underwent both viral testing and pneumococcal density determinations (Appendix Figure 1). Relevant demographic characteristics of study children are shown in the Table. Pneumococcus was detected in 566/849 (67%) nasopharyngeal samples from asymptomatic children. At least 1 respiratory virus was detected in 357/849 (42%) samples from asymptomatic children, most commonly HRV (31%) and AdV (11%), whereas detections of influenza, MPV, PIV, and RSV in asymptomatic children were uncommon (<3%), as previously reported (*23*). 

**Figure 1:**
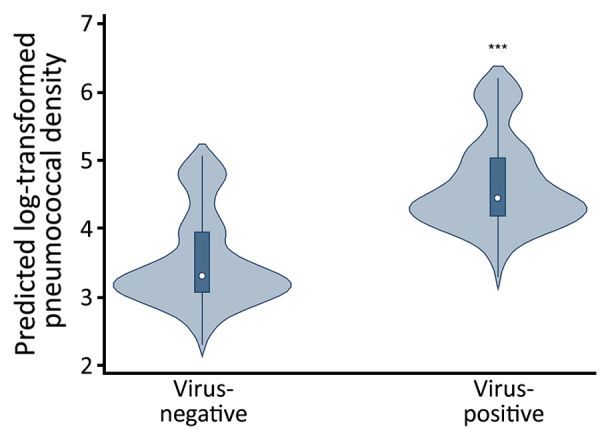
Violin plots of predicted log_10_-transformed pneumococcal colonization densities by any viral detection among children <3 years of age, Respiratory Infections in Andean Peruvian Children study, May 2009–September 2011. Predicted densities were estimated from the final multivariable linear quantile mixed effects model. Circles indicate median densities, boxes represent interquartile range, lines extend through the upper and lower adjacent values, and the density plot width indicates the predicted frequency of observations. ***p<0.001.

**Table:** Selected demographic characteristics of 480 children from whom nasopharyngeal samples were obtained during asymptomatic periods, Respiratory Infections in Andean Peruvian Children study, May 2009–September 2011*

Characteristic	Value
Sex	
M	253 (53)
F	227 (47)
Median age at enrollment (IQR), mo	7.7 (1.0–18.8)
No. samples per child (IQR)	2 (1–2)
No. persons per household (IQR), n = 476	5 (4–6)
No. children age <5 y in household, n = 476	
1	323 (68)
2	140 (29)
>3	13 (3)
Shares a bed, n = 473	461 (97)
Received >1 PCV7 dose, n = 468	262 (56)

*Values are no. (%) children unless indicated otherwise. IQR, interquartile range; PCV7, 7-valent pneumococcal conjugate vaccine.

Co-detection of >1 respiratory virus occurred in 48/849 (6%) samples. HRV was present in 40/48 (83%) co-detections, most commonly in combination with AdV, with or without an additional virus (36/40 [90%]). Colonization with pneumococcus was more common in samples in which >1 respiratory virus was detected (280/357 [78%]) than in those in which a respiratory virus was not detected (286/492 [58%]) (p<0.001). Rhinorrhea was reported in 219/357 (61%) children in whom a virus was detected, compared with 227/492 (46%) in whom a virus was not detected (p<0.001). At least 1 respiratory virus was detected in 219/446 (49%) children with rhinorrhea, compared to 138/403 (34%) without rhinorrhea. Antibiotic exposure preceding collection of each asymptomatic nasopharyngeal sample was uncommon, occurring in only 12/849 (1%) samples (antibiotic exposure was unknown in 9/849 samples [1%]).

### Pneumococcal Densities and Associated Factors

In unadjusted comparisons, log_10_-transformed pneumococcal densities were higher during asymptomatic periods when >1 respiratory virus was detected (median 4.95 [interquartile range (IQR) 3.11–6.35]) than when no respiratory viruses were detected (median 3.35 [IQR 0–4.95]; p<0.001). When densities were examined according to detection of specific viruses, densities differed significantly by viral group (p<0.001); the highest densities were observed in the HRV-only group. In Bonferroni-adjusted pairwise median comparisons, pneumococcal densities were significantly higher when HRV only (median 5.18 [IQR 3.40–6.43]; p<0.001) or viral co-detections (median 4.71 [IQR 3.46–6.35]; p = 0.003) were present compared with samples that were virus-negative (3.35 [IQR 0–4.95]). In Bonferroni-adjusted median comparisons, pneumococcal density from samples with AdV-only infection (median 4.39 [IQR 3.15–6.08]) was not significantly different from that from samples with no respiratory viruses detected (p = 0.087).

In the multivariable mixed effects quantile regression model, pneumococcal densities during asymptomatic periods were not associated with age, sex, antibiotic exposure, or history of pneumococcal vaccination. However, detection of any (>1) virus was significantly associated with higher log_10_-transformed pneumococcal densities (Appendix Table 1). The addition of rhinorrhea, a common manifestation of viral infections, to the model attenuated the observed association between viral infections and pneumococcal density, but the association remained statistically significant. 

**Figure 2:**
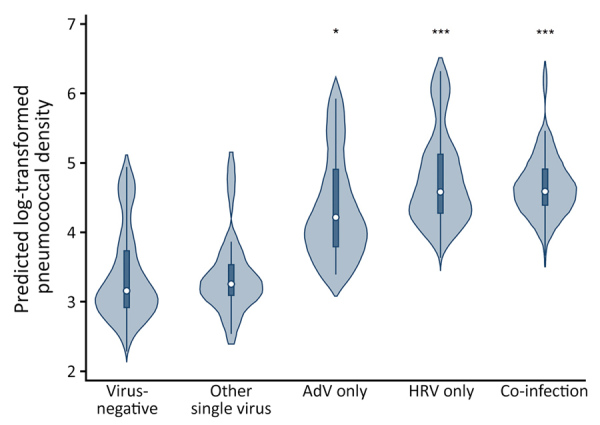
Violin plots of predicted log_10_-transformed pneumococcal colonization densities by detection of specific viruses among children <3 years of age, Respiratory Infections in Andean Peruvian Children study, May 2009–September 2011. Predicted densities were estimated from the final multivariable linear quantile mixed effects model. Circles indicate median densities, boxes represent interquartile range, lines extend through the upper and lower adjacent values, and the density plot width indicates the predicted frequency of observations. *p<0.05; ***p<0.001. AdV, adenovirus; HRV, rhinovirus.

We compiled violin plots of predicted pneumococcal densities according to detection of any respiratory virus as estimated from the multivariable linear quantile mixed effects model (Figure 1). When specific viruses were examined in multivariable analyses, detection of HRV only, detection of AdV only, or co-detection of >1 virus was significantly associated with increased pneumococcal densities compared with densities in samples that were virus-negative. We observed no significant association between detection of other less frequently detected viruses (RSV, PIV, influenza, MPV) and pneumococcal densities (Appendix Table 2). As in the main analysis, the addition of rhinorrhea to the model slightly attenuated, but did not eliminate, the observed associations. We compiled predicted densities associated with specific viral infections as estimated from the multivariable linear quantile mixed effects model (Figure 2).

### Exploration of Asymptomatic Pneumococcal Densities and Risk for Subsequent ARI

**Figure 3:**
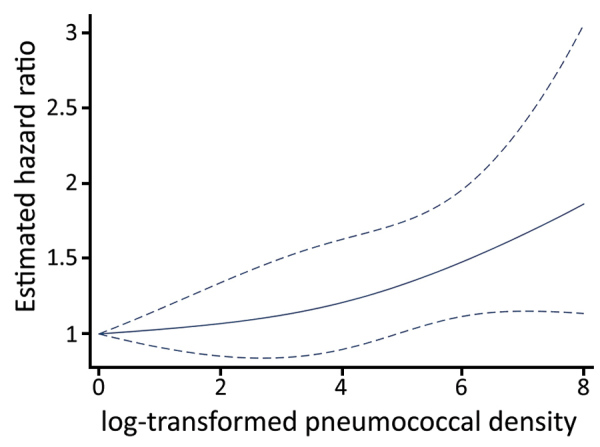
Association between asymptomatic pneumococcal densities and risk of subsequent acute respiratory illness among children <3 years of age, Respiratory Infections in Andean Peruvian Children study, May 2009–September 2011. Estimated hazard ratios correspond to comparisons of increasing log_10_-transformed pneumococcal density relative to the lowest detectable densities (p = 0.013). Solid lines represent the point estimates for the hazard ratio by log-transformed pneumococcal density; dashed lines represent 95% CIs. Estimates were obtained from a frailty model that adjusted for age, sex, month, prior antibiotic exposure, viral detection, and pneumococcal conjugate vaccination status. Pneumococcal densities were modeled by using restricted cubic splines to allow examination of nonlinear associations.

We explored the association of pneumococcal density during asymptomatic periods and the time to next ARI by using log-transformed pneumococcal density as the exposure of interest and ARI as the outcome in a frailty model. Compared with the lowest pneumococcal density, increases in pneumococcal density were significantly associated with increased incidence of subsequent ARI (Figure 3; Appendix Table 3) (p<0.001). Younger age, receipt of >1 PCV7 dose, and detection of >1 respiratory virus during asymptomatic periods were significantly associated with lower incidence of ARI. In addition, sample collection during the middle months of spring (October and November) was associated with lower incidence of subsequent ARI. Similar findings were observed when rhinorrhea was included in the model instead of viral detection (Appendix Table 4, Figure 2), suggesting that the association between viral detection and ARI might be at least partially mediated by rhinorrhea.

## Discussion

Our findings demonstrate that viral detections during asymptomatic periods are associated with increases in nasopharyngeal pneumococcal colonization density, and further, that higher pneumococcal density during asymptomatic periods is associated with subsequent onset of an ARI. These findings expand upon earlier observations from our group and others that increases in nasopharyngeal pneumococcal colonization density are associated with symptomatic respiratory illness and pneumonia and with the detection of respiratory viruses during ARI periods (*6*,*7*,*14*,*15*). We now report that those associations are not restricted to periods of symptomatic disease and demonstrate an increased risk for ARI with increasing pneumococcal density during asymptomatic periods as well. Of interest, increased pneumococcal density was associated with the risk for subsequent ARI in a nonlinear manner and appeared particularly evident at higher pneumococcal densities (>10^4^). These findings suggest that asymptomatic viral infection during asymptomatic periods might present colonizing pneumococci with an opportunity to expand.

Most ARI episodes in our study were mild, self-limited without antibiotic use, and associated with respiratory virus detection (*28*). Our finding that increases in pneumococcal density during asymptomatic episodes were associated with increased likelihood of subsequent onset of these mostly mild, viral ARI might suggest a contributing role of colonizing pneumococci in these illnesses. Of note, this association only became significant at the highest levels of pneumococcal density. A previous study of young children with nasopharyngeal pneumococcal colonization indicated that higher colonization density was associated with high levels of markers of nasopharyngeal inflammation compared with children with lower-density colonization (*44*). We postulate that increased nasopharyngeal inflammation from increased pneumococcal density might facilitate viral infection of an exposed susceptible person. The hypothesis that nasopharyngeal pneumococcal colonization patterns and density might influence subsequent onset of nonpneumococcal ARI is also consistent with our observation that children who had received >1 PCV vaccination had a reduced risk for ARI following asymptomatic viral detection compared with unvaccinated children.

A recent study by DeMuri et al. (*27*) examined colonization density with pneumococci and other respiratory bacteria during asymptomatic periods in children 4–7 years of age. They found that the densities of pneumococcus, *Moraxella catarrhalis*, and *Haemophilus influenzae* all increased when respiratory viruses were detected, although the study design did not allow determination of subsequent ARI risk (*27*). In that study, the association of viral detection and nasopharyngeal bacterial density was strongest with pneumococcus; pneumococcal densities approached those observed during periods of viral upper respiratory illness. HRV, coronaviruses, and viral co-detections (≥1 virus present) were also significantly associated with higher densities than when no virus was detected. Detection of AdV in their patients was not significantly associated with increased pneumococcal densities during asymptomatic periods, but those observations were limited to only 4 AdV-positive samples.

Although both viral detection and rhinorrhea were associated with increased pneumococcal density, both were also associated with a lower incidence of subsequent ARI. Specifically, children with viral infection or rhinorrhea had a lower incidence of subsequent ARI than children without viral infection or rhinorrhea. The mechanism of this association is unclear but might involve the phenomenon of viral interference, in which infection with 1 virus prevents or mitigates infection with a different virus, perhaps through the induction of proinflammatory cytokines or other immune mediators (*41*), because the presence of rhinorrhea might indicate a viral infection with a virus detected or not detected in our study. Rhinitis has also been shown to occur after intranasal inoculation of pneumococcus in healthy adults and in mouse models (*45*), and nasal discharge has been associated with pneumococcal detection even in the absence of respiratory viral infection (*46*), suggesting that an inflammatory response might be an important component of early pneumococcal colonization, which might have important host consequences. Furthermore, several studies have also demonstrated the role of normal airway mucus and nasal secretions in lining epithelial surfaces and trapping and removing pathogens from the airway through mucociliary clearance (*47*,*48*), potentially providing an effective barrier to acquisition of new respiratory viruses, bacteria, or both.

In spite of the strengths of our longitudinal design, our study has several limitations. Because detection of viruses other than HRV and AdV during asymptomatic periods was uncommon in our cohort, the power to detect associations with these other specific viruses and pneumococcal densities was low. HRV was detected in >80% of samples in which >1 virus was detected, limiting inferences about co-detections with other viruses. Because of enhanced sensitivity of rRT-PCR for detection of respiratory viruses relative to culture, it might be difficult to interpret the clinical significance of detection of HRV and AdV by PCR in asymptomatic children (i.e., whether these detections might represent prolonged shedding from a prior illness or a recent asymptomatic infection). The study was conducted in a rural region in which the incidence of pneumonia is higher than national rates (*28*,*49*). However, incidence of other common respiratory diseases, such as otitis media, sinusitis, and other types of respiratory illnesses, was not available. Highest densities were observed during the 2009 mid-spring season, but examination of several consecutive seasons would be useful to clarify the role of seasonality in our observations. In our assessment of the role of colonization density during asymptomatic periods on the risk for subsequent ARI, we did not systematically assess for pneumococcal colonization or density during the subsequent ARI, so direct density comparisons were not possible between the asymptomatic and subsequent ARI periods. Furthermore, we must note that several factors might play a role in increases in pneumococcal colonization density. A recent study from Kenya found that increases in density associated with viral detection were modest relative to the baseline variation in density in individual patients (*50*) and postulated that other factors, such as the host immune response to previous viral infections (*6*,*7*,*27*), also play an important role in the association between high levels of pneumococcal density and risk for subsequent ARI. However, the potential influence of immune, genetic, or other environmental factors on subsequent ARI risk was not measured in our study. Also, the associations observed with pneumococcal density might be serotype-specific, but serotype information was not available for those samples from patients with subsequent ARI. Furthermore, interactions between pneumococcus and other colonizing nasopharyngeal bacteria might play an important role in the host response to pneumococcal acquisition as well as the dynamics of pneumococcal density and pathogenesis (*29*), but associated changes in the nasopharyngeal microbiome were not assessed in this study. The presence of an asymptomatic viral infection was associated with a lower risk for subsequent symptomatic ARI in this study. Although we postulate that such a relationship might have a time-limited impact, our study was set up with a short follow-up time and was not designed to determine the duration of this association. Additional studies that have a longer follow-up time and are specifically designed to determine the temporality of the observed association would be useful. Future studies will assess the relationship between viral detection and serotype and density patterns over time in individual patients, along with associated changes in the respiratory microbiome during both asymptomatic and ARI periods. Finally, our study did not assess transmission patterns of colonizing pneumococci. Whether the increase in densities associated with viral detection have an impact on transmission is unclear.

In summary, we found that viral detections during asymptomatic periods are associated with increases in nasopharyngeal pneumococcal colonization density. Furthermore, we found that pneumococcal density, especially at high levels, is associated with subsequent development of ARI in young children in Peru. These findings suggest that interactions between viruses and pneumococci in the nasopharynx during asymptomatic periods might have a role in onset of subsequent ARI.

Appendix Additional information about nasopharyngeal pneumococcal density during asymptomatic respiratory virus infection and risk for subsequent acute respiratory illness.
