# Supplementary material for: Nasopharyngeal Pneumococcal Density during Asymptomatic Respiratory Virus Infection and Risk for Subsequent Acute Respiratory Illness
Source: Emerg Infect Dis. 2019 Nov;25(11):2040–7. doi: 10.3201/eid2511.190157 (PMC6810199; doi:10.3201/eid2511.190157)
Supplement: Appendix — Additional information about nasopharyngeal pneumococcal density during asymptomatic respiratory virus infection and risk for subsequent acute respiratory illness. [file 19-0157-Techapp-s1.pdf]

Article DOI: https://doi.org/10.3201/eid2511.190157

Nasopharyngeal Pneumococcal Density during Asymptomatic Respiratory Virus Infection and Risk for Subsequent Acute Respiratory Illness

Appendix

Appendix Table 1. Multivariable mixed effects quantile regression of the association between detection of any virus and pneumococcal density during asymptomatic periods*

Characteristic	Coefficient	SE	LL 95%	UL 95%	p-value
(Intercept)	3.05	0.42	2.22	3.89	**<0.001**
Any virus positive	1.15	0.26	0.64	1.66	**<0.001**
Age	0.12	0.14	–0.17	0.40	0.423
Female sex	0.24	0.23	–0.22	0.70	0.305
Any PCV7 dose	0.02	0.31	–0.60	0.63	0.963
June	–0.29	0.29	–0.86	0.28	0.310
July	–0.83	0.50	–1.81	0.15	0.098
August	–0.40	0.34	–1.07	0.27	0.238
September	–0.12	0.36	–0.83	0.60	0.750
October	1.44	0.41	0.62	2.26	**<0.001**
November	0.59	0.40	–0.20	1.38	0.144
Antibiotics yes	–0.18	0.75	–1.67	1.32	0.815
Antibiotics unknown	–0.53	0.97	–2.46	1.40	0.585

* p-value <0.05 considered statistically significant; indicated in bold text.

Appendix Table 2. Multivariable mixed effects quantile regression of the association between detection of specific viruses and pneumococcal during asymptomatic periods*

Characteristic	Coefficient	SE	LL 95%	UL 95%	p-value
(Intercept)	2.92	0.38	2.1757	3.67	**<0.001**
Other single virus	0.21	0.54	–0.86	1.27	0.703
AdV only	0.98	0.39	0.20	1.77	**0.014**
HRV only	1.40	0.25	0.91	1.88	**<0.001**
Co-infection	1.45	0.38	0.71	2.20	**<0.001**
Age	0.16	0.13	–0.11	0.42	0.250
Female sex	0.23	0.24	–0.25	0.70	0.344
Any PCV7 dose	0.01	0.30	–0.59	0.62	0.961
June	–0.41	0.28	–0.97	0.15	0.152
July	–0.72	0.51	–1.73	0.29	0.162
August	–0.41	0.33	–1.06	0.24	0.213
September	–0.20	0.36	–0.92	0.51	0.575
October	1.36	0.40	0.56	2.16	**0.001**
November	0.46	0.40	–0.33	1.24	0.251
Antibiotics yes	–0.10	0.78	–1.65	1.45	–0.894
Antibiotics unknown	–0.35	0.88	–2.10	1.40	–0.690

* p-value <0.05 considered statistically significant; indicated in bold text.

Appendix Table 3. Multivariable survival frailty model of the association between log-transformed pneumococcal density and viral detection, and subsequent ARI censored at 60 d and excluding rhinorrhea as a covariate*

Characteristic	Coefficient	Exponentiated coefficient (Hazard ratio)	LL 95%	UL 95%	p-value
Any virus positive	−0.31	0.73	0.56	0.95	**0.02**
Age	−0.33	0.72	0.62	0.84	**<0.001**
June	0.13	1.14	0.80	1.63	0.480
July	0.04	1.05	0.57	1.91	0.880
August	0.49	1.64	0.99	2.72	0.057
September	−0.30	0.74	0.45	1.24	0.260
October	−0.73	0.48	0.29	0.73	**0.005**
November	−0.68	0.51	0.31	0.82	**0.006**
Antibiotics yes	0.61	1.85	0.73	4.65	0.190
Antibiotics unknown	0.43	1.54	0.56	4.23	0.400
Any PCV7 dose	−0.41	0.66	0.47	0.94	**0.022**
Female sex	−0.15	0.86	0.66	1.12	0.260

*p-value <0.05 considered statistically significant; indicated in bold text. Pneumococcal densities were transformed using restricted cubic splines, and for ease of interpretation their effects on the risk of subsequent ARI are not listed in table but represented in Figure 3 (p-value for pneumococcal density is 0.031).

Appendix Table 4. Multivariable survival frailty model of the association between log-transformed pneumococcal density and rhinorrhea, and subsequent ARI censored at 60 d and excluding viral detection as a covariate*

Characteristic	Coefficient	Exponentiated coefficient (Hazard ratio)	LL 95%	UL 95%	p-value
Age	−0.28	0.76	0.65	0.87	**<0.001**
June	0.10	1.10	0.78	1.56	0.577
July	−0.10	0.91	0.51	1.61	0.737
August	0.36	1.43	0.88	2.33	0.144
September	−0.36	0.70	0.43	1.15	0.158
October	−0.73	0.48	0.30	0.79	**0.004**
November	−0.67	0.51	0.32	0.81	**0.005**
Antibiotics yes	0.47	1.61	0.67	3.86	0.289
Antibiotics unknown	0.34	1.41	0.54	3.66	0.486
Rhinorrhea	−0.715	0.49	0.38	0.63	**<0.001**
Any PCV7 dose	−0.40	0.67	0.48	0.93	**0.017**
Female sex	−0.13	0.88	0.69	1.12	0.283

*p-value <0.05 considered statistically significant; indicated in bold text. Pneumococcal densities were transformed using restricted cubic splines, and for ease of interpretation their effects on the risk of subsequent ARI are not listed in table but represented in Appendix Figure 1 X (p-value for pneumococcal density is 0.02).

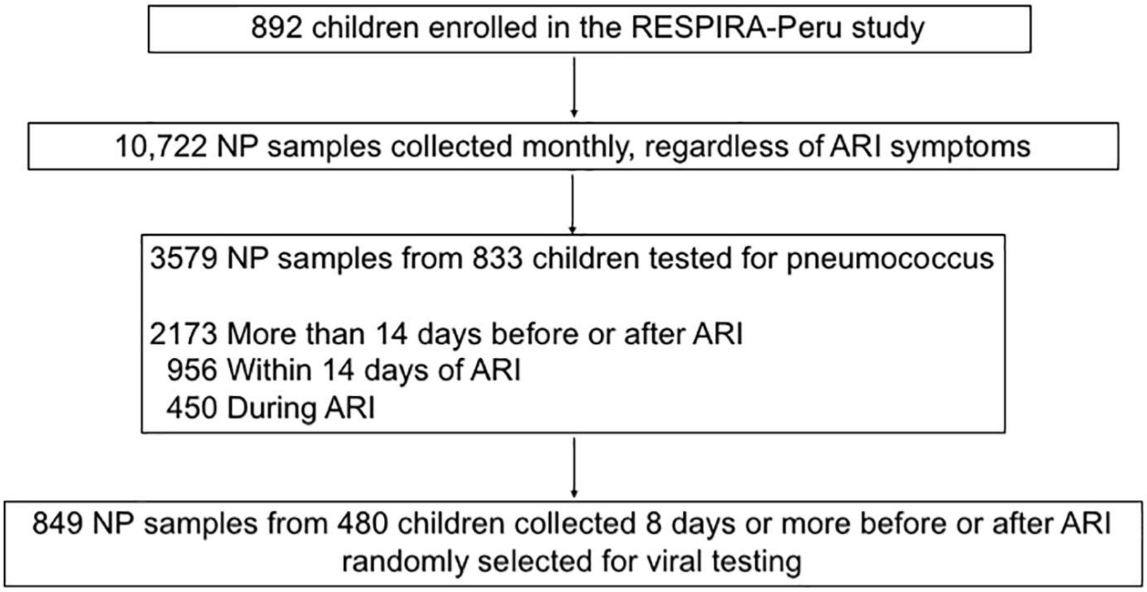

Appendix Figure 1. Flow diagram outlining RESPIRA-Peru study enrollment, sample collection, and sample selection for this analysis.

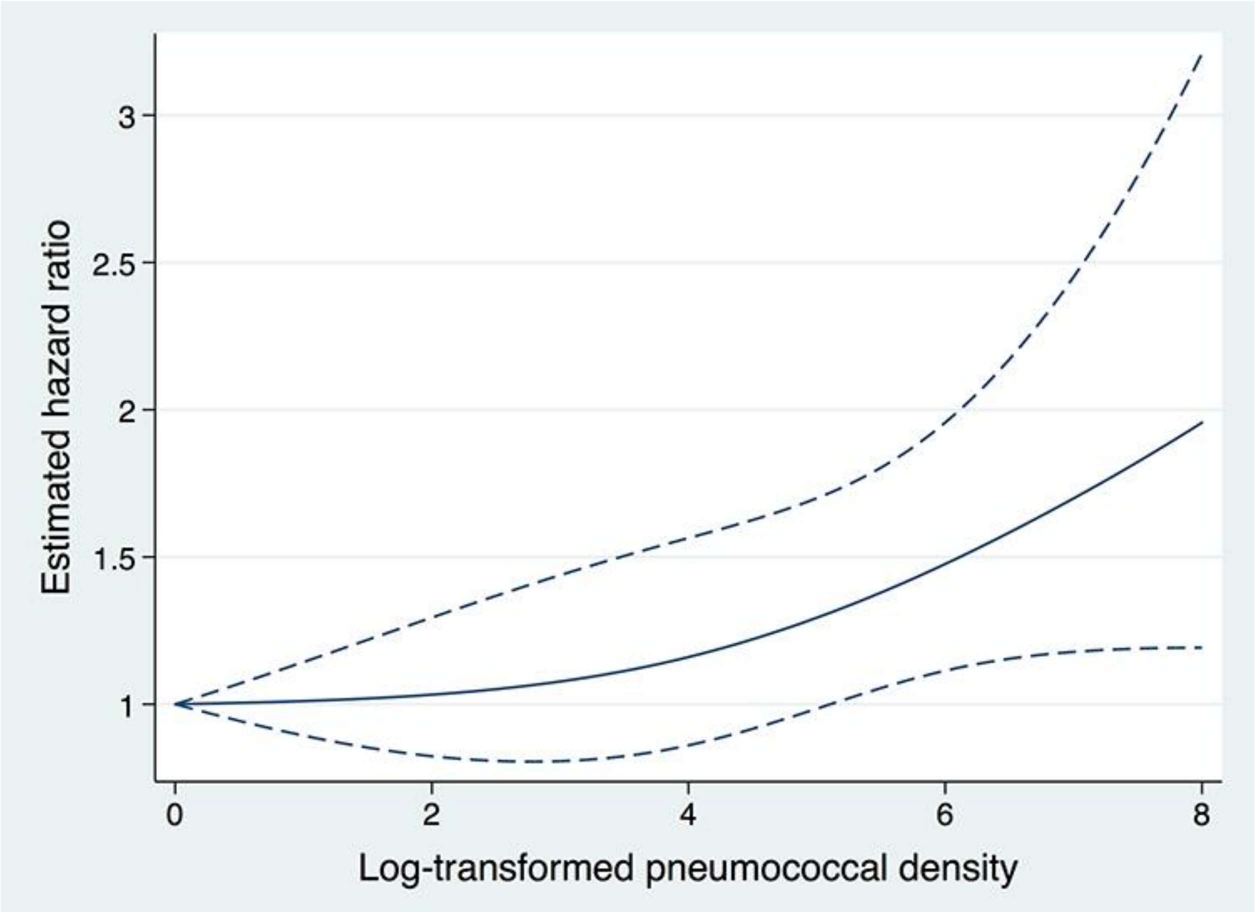

Appendix Figure 2. Association between asymptomatic pneumococcal densities and risk of subsequent ARI. Estimated hazard ratios correspond to comparisons of increasing log-transformed pneumococcal density relative to the lowest detectable densities ($p = 0.008$). Solid lines represent the point estimates for the hazard ratio by log-transformed pneumococcal density; dashed lines represent 95% confidence intervals for the hazard ratio. Estimates were obtained from a frailty model that adjusted for age, sex, month, prior antibiotic exposure, rhinorrhea, and PCV vaccination status. Pneumococcal densities were modeled using restricted cubic splines to allow examination of nonlinear associations.
